# Spontaneous Rupture of Empyema Necessitans in the Emergency Department

**DOI:** 10.7759/cureus.14822

**Published:** 2021-05-03

**Authors:** Andrew Crouch, Johnson Lay, Arianna Neeki, Fanglong Dong, Michael Neeki

**Affiliations:** 1 Emergency Medicine, Arrowhead Regional Medical Center, Colton, USA; 2 Anatomy, California University of Science and Medicine, Colton, USA; 3 Emergency Medicine, California University of Science and Medicine, Colton, USA

**Keywords:** empyema, empyema necessitans, rupture of empyema, computerized tomography, thoracostomy tube

## Abstract

An empyema necessitans is a rare complication of a collection of purulent material in the pleural space that spreads outside of the pleural cavity and involves the soft tissues of the chest wall. Due to compression forces created by the size of the collection of empyema in the chest cavity, patients are usually symptomatic and present with severe dyspnea. Chest X-ray or ultrasound of the chest cavity are the ideal screening tools to visualize the empyema and followed by computerized tomography scan of the chest to confirm the presence and extent of the pathology. In rare occasions, the empyema can rupture spontaneously, which may lead to critical situation requiring emergent intervention. We report the case of a 72-year-old male who presented to the emergency department with severe dyspnea and was diagnosed with empyema necesitans. During the initial management of the case, the empyema necessitans ruptured spontaneously and required emergent interventions to stabilize the patient.

## Introduction

Empyema is described as one of the subtypes of pleural effusion due to bacterial infections in the chest cavity [1]. It is defined as pus in the pleural space and classified as complicated parapneumonic effusion [1-3]. Factors can be used to predict the probability of development of empyema in patients admitted with community-acquired pneumonia; for example, history of alcoholism and intravenous drug use has been associated with an increased risk, while history of chronic obstructive pulmonary disease was associated with a decreased risk [2].

Falguera et al. noted relevant baseline clinical characteristics such as age <60 years, alcoholism, pleuritic pain, tachycardia >100 beats/minute, and leukocytosis that could predict the development of empyema [3]. In addition, they found a higher incidence of anaerobes and Gram-positive cocci (mainly *Streptococcus pneumoniae*, *Staphylococcus aureus*, and *Streptococcus viridans*) as the causative microorganisms [3]. The diagnosis of empyema relies on imaging studies such as chest X-ray or ultrasonography as the screening tool followed by computerized tomography (CT) scan of the chest which have greater sensitivity for fluid detection and further help determine the extent of the pleural space infection [4].

Empyema can result in a rare complication called empyema necessitans, where the pleural infection spreads outside of the pleural space and involves the soft tissues of the chest wall [5,6]. The cause of this complication can have various etiologies that include trauma to the region, recent thoracotomy, and necrotizing pneumonia [7]. It may burst out through a structurally weak part of the chest wall to the skin and release pus to the surrounding structures in any direction [8]. The causative organisms for this complication have been predominantly *Mycobacterium tuberculosis* and *Actinomyces israelii* [9-11]. However, there have been case reports that describe a variety of other etiologies, including *Staphylococcus aureus*, *Aspergillus* spp., *Streptococcus pneumoniae*, *Aggregatibacter actinomycetemcomitans*, and *Streptococcus agalactiae* [6,8,12-15]. We report the case of a 72-year-old male who presented to the emergency department (ED) in severe distress and was diagnosed with empyema.

## Case presentation

A 72-year-old male was transported by Emergency Medical Services to the ED with severe shortness of breath and altered mental status. His past medical history was significant for hypertension, benign prostatic hypertrophy, and mesothelioma. The mesothelioma had been treated with lobe resection on the right few years prior to this visit (unable to obtain the exact time from the patient’s charts) and the patient underwent chemotherapy. Additionally, he had been treated for a pleural effusion on the right side three months prior to this event.

**Figure 1:**
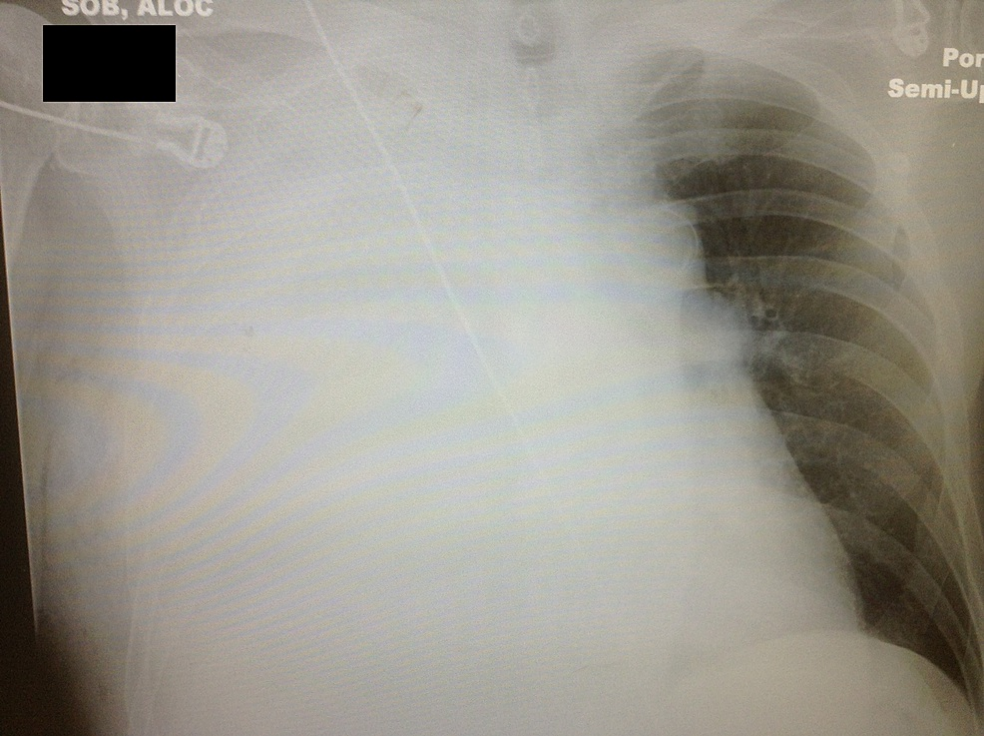
Chest X-ray at the time of arrival.

**Figure 2:**
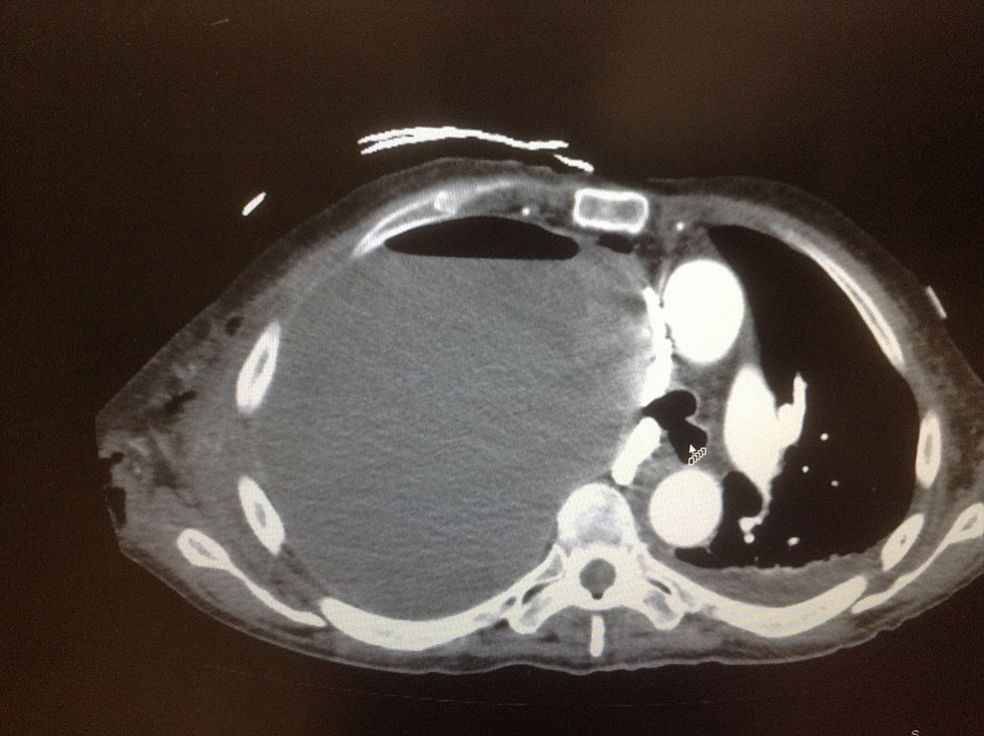
CT scan of the chest cavity. The arrow indicates one of the air-fluid level in the empyema necessitans. CT: computerized tomography

The patient presented with an initial blood pressure of 85/50 mmHg, heart rate of 110 beats/minute, and respiratory rate of 34 breaths/minute. He was hypoxic with oxygen saturation of 85% on room air. On the physical examination, he was noted to have absent breath sounds on the right side of the chest. A 5-cm area of fluctuance and subcutaneous air was noted over the lateral right chest wall. Chest X-ray was done shortly after arrival to the ED which showed complete opacification of the right hemithorax and left-sided deviation of the mediastinal content (Figure 1). After the initial resuscitation with 1,000 mL of intravenous (IV) normal saline infusion along with supplemental oxygen via non-rebreather face mask, the patient’s condition improved and he subsequently underwent CT scan of his chest with IV contrast. The CT scan revealed a fluid distention of the right hemithorax with an air-fluid level approximately 14 cm across. Multiple pockets of air and fluid were noted extending through the chest wall to the subcutaneous tissues. Additionally, the heart and mediastinal structures were shifted to the left (Figure 2).

**Figure 3:**
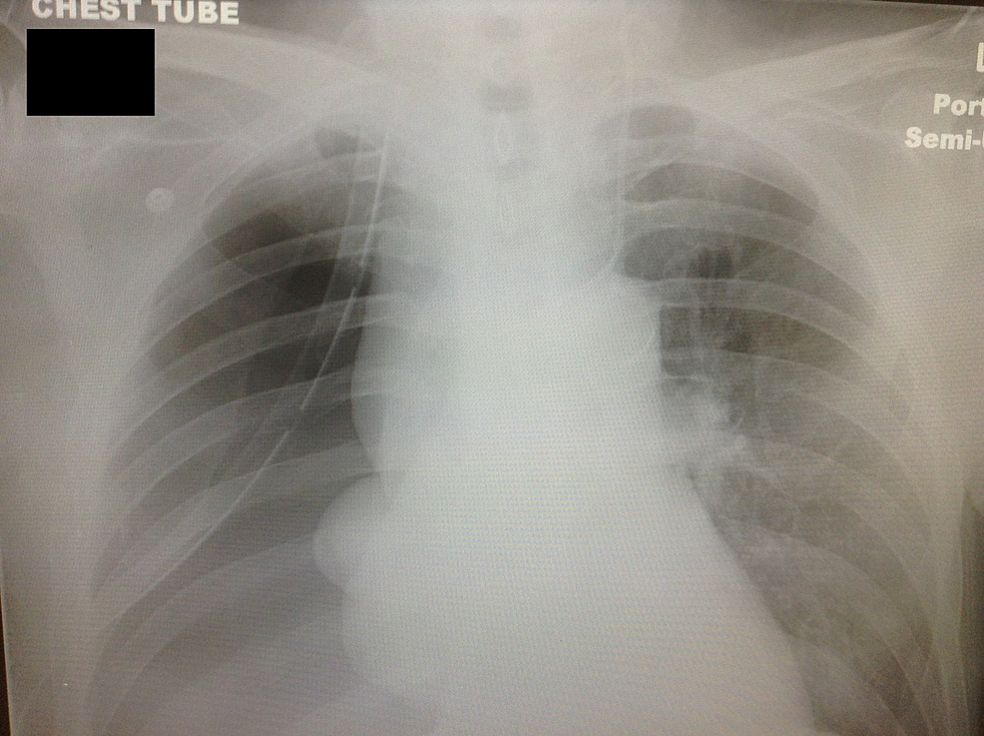
Chest X-ray following thoracostomy tube placement.

Following the return from the CT scan, the fluid collection on the right chest wall ruptured spontaneously while moving the patient from stretcher to his ED bed. A large volume of foul-smelling, purulent material and air began to quickly exit through the wound opening. Immediate placement of a 36-French thoracostomy tube superior to the preexisting lesion decompressed an additional 1,800 mL of purulent fluid. A follow-up chest X-ray was taken to confirm the placement of the thoracostomy tube and reevaluate the chest cavity. Shortly after the thoracostomy tube placement, his oxygen requirement drastically decreased and shortly thereafter his blood pressure improved (Figure 3). The patient was admitted to the intensive care unit on parenteral antibiotics. Pathological analysis of the purulent fluid sample confirmed findings consistent with empyema with no signs of malignancy. Cultures from the blood and purulent fluid were negative possibly due to insufficient time to grow and detect any microbes. Following a 16-day course of hospital stay, the patient was discharged to an extended care facility.

## Discussion

An empyema is a collection of purulent material in the pleural space and is typically the result of pneumonia or lung abscess. The presence of empyema is associated with a high mortality rate of 5.4% to 22%, depending on the patient’s risk factors [16]. Empyema necessitans is a rare condition in which an empyema extends into the soft tissues of the thorax after eroding through the parietal pleural. The purulent material can eventually rupture through the skin to spontaneously drain the empyema. This condition is rare and carries a high mortality. Organisms that can cause empyema necessitans include *Mycobacterium tuberculosis*, *Actinomyces* spp., *Streptococcus* spp., *Staphylococcus aureus*, *Fusobacterium* spp., and polymicrobial infections. Cultures and Gram stain are often falsely negative after patients had been pretreated with antibiotics [16].

In this case, on the initial chest X-ray, mediastinal deviation away from the empyema was noted; hence, a “tension pneumo-empyema.” Following the patient’s lobectomy and pneumonectomy procedures, the empty space was slowly filled by serous fluid and the opposite lung expanded, causing structures to shift toward the surgical site. This finding can be normal in patients who have undergone prior lung resection surgeries. Deviation of midline structures away from the surgical site represents a volume expanding process and a sign of complications. Differential diagnoses in these cases can include recurrent neoplasm, empyema, hemothorax, and chylothorax [17].

In this particular case, diagnosis of empyema was determined based on initial screening with chest X-ray followed by CT scan to visualize the extent of this pleural space involvement. The initial chest X-ray showed complete opacification of the right hemithorax and left-sided deviation of the mediastinum. Standard chest X-rays allow for visualization of the entire pleural space, underlying lung, mediastinum, chest wall, and spine for potential causes and complications of the pleural process [4]. In the context of empyema, infected pleural fluid collections are visualized with chest X-ray imaging; however, volume and viscosity of the pleural fluid, the patient’s position, and presence of pleural loculations dictate the overall appearance [4]. Therefore, CT scan of the chest cavity is necessary to reveal important details and the extent of the pathology in the chest cavity. This case demonstrated the advantages and sensitivity of the CT scan diagnosis of empyema. This is in agreement with Heffner et al. who noted that if left untreated, empyemas can progress to a fibrothorax with pleural peels that appear as uniform smooth thickenings of the pleurae [4]. Furthermore, it can may lead to hypertrophy of the extrapleural fat and reduced volume of the affected hemothorax with narrowing of the intercostal spaces and shift the mediastinum to the affected side [4]. As a result, CT scan of the thorax can go beyond the view of a standard chest X-ray by visualizing the detail and volume of the fluid collection and providing a better clinical picture.

There are multiple accounts in the literature of cardiac arrest occurring from rapidly expanding empyema leading to tension pyothorax [17,18]. The rapidly expanding empyema compresses the major vascular structures, which results in decreased venous return and lung volume. In two similar case reports, the pyothorax was treated by emergency thoracostomy tube placement [19,20]. Although the current case more closely resembles a chronic expanding empyema with a resulting mass effect and sepsis, it is important to note that a tension pyothorax and pneumothorax can occur as the volume of the empyema increases in the chest cavity. The management and care of these patients by the ED team has to be quick in reaction to this very critical and yet rare phenomena.

## Conclusions

An empyema necessitans is a rare complication of pleural cavity infection and emergency physicians should be vigilant about the patient’s history and risk factors, which can weaken the chest wall during initial care in the ED. Further suggestions to consider include closely monitoring the patient, being prepared to place a chest tube, being ready for acute decompensation, administering early IV antibiotics, and having surgery on board early. Providing such patient care and interventions is essential to improving patient outcomes.
